# ABS–Scan: *In silico *alanine scanning mutagenesis for binding site residues in protein–ligand complex

**DOI:** 10.12688/f1000research.5165.2

**Published:** 2014-12-01

**Authors:** Praveen Anand, Deepesh Nagarajan, Sumanta Mukherjee, Nagasuma Chandra

**Affiliations:** 1 Department of Biochemistry, Indian Institute of Science, Bangalore, 560012, India; 2 IISc Mathematics Initiative, Indian Institute of Science, Bangalore, 560012, India

## Abstract

Most physiological processes in living systems are fundamentally regulated by protein–ligand interactions. Understanding the process of ligand recognition by proteins is a vital activity in molecular biology and biochemistry. It is well known that the residues present at the binding site of the protein form pockets that provide a conducive environment for recognition of specific ligands. In many cases, the boundaries of these sites are not well defined. Here, we provide a web-server to systematically evaluate important residues in the binding site of the protein that contribute towards the ligand recognition through in silico alanine-scanning mutagenesis experiments. Each of the residues present at the binding site is computationally mutated to alanine. The ligand interaction energy is computed for each mutant and the corresponding ΔΔG values are calculated by comparing it to the wild type protein, thus evaluating individual residue contributions towards ligand interaction. The server will thus provide a ranked list of residues to the user in order to obtain loss-of-function mutations. This web-tool can be freely accessed through the following address: http://proline.biochem.iisc.ernet.in/abscan/.

## Introduction

Currently (as of April 3, 2014) ^1^ there exist more than 72000 experimentally determined protein structures complexed with small molecule ligands, providing an extensive data resource on protein binding sites. These binding sites vary in size ranging from six to thirty residues depending upon the size and the nature of the ligand. In most cases, the contribution of the individual amino acids towards the binding of a given ligand is not well understood. A well-established method of demonstrating the importance of a residue at the site is to create point mutants through site-directed mutagenesis ^2^. Efforts towards characterization of entire functional site include tools such as alanine scanning mutagenesis (ASM) ^3^ where each residue is mutated to an alanine and its effect on the function is evaluated. ASM is indeed a well-used technique in experimental biology and has been successfully applied to the problems of protein folding and stability ^4^, protein-protein ^5, 6^, and protein-ligand ^7^ interactions. The experimental success of this technique has resulted in further developments, including high-throughput and low-cost variants ^8^, greatly expanding its reach. Yet, given the time, cost and effort required for carrying out experimental biochemistry, a large majority of proteins are yet to be studied through this method.

Due to availability of a variety of structural bioinformatics tools, it is now feasible to carry out alanine scanning mutagenesis computationally ^9^. Spurred by the successes and widespread adoption of the ASM technique, various computational resources now exist for *in-silico* alanine scanning. Prominent examples include Modeller ^10^ and the Rosetta software suite ^11^. However, most packages are command-line oriented and are out of reach for researchers. Alanine scanning webservers with intuitive user interfaces such as Robetta webserver ^12^, the Rosetta Design web-server ^13^, ROSIE ^14^, FOLDX ^15^, BeATMuSiC ^16^, DrugScore ^PPI^ ^17^ exist for the problems of protein folding, protein stability and protein-protein interactions. Although, there are workflows to evaluate ligand-binding energetics which require significant computational time and setup through free-energy calculations involving Molecular Mechanics/Generalized Born Surface Area method (MM-GBSA) ^18– 20^, there is however, no intuitive web-tool available for analyzing alanine-scanning mutations of small-molecule binding site residues in real time. A common requirement for an experimental biochemist is to identify which amino acids to mutate in the protein to generate loss-of-function mutants. A web-tool to cater to that specific need will therefore be highly useful. The analysis will also provide deep insights into critical residues for interaction, residue pairs or sets that when mutated will abolish ligand binding and provide analytical insights for lead refinement in the process of drug discovery, as well as understand drug resistance due to mutations.

We present a computational workflow and webserver, Alanine Binding Site-Scan (ABS-Scan), for automated alanine-scanning mutagenesis of protein-ligand interface residues. The workflow combines the libraries of widely used software packages including Modeller ^10^ for site-specific alanine mutagenesis and Autodock ^21^ for energetic evaluation of protein-ligand complexes.

## Workflow

**Figure 1.**
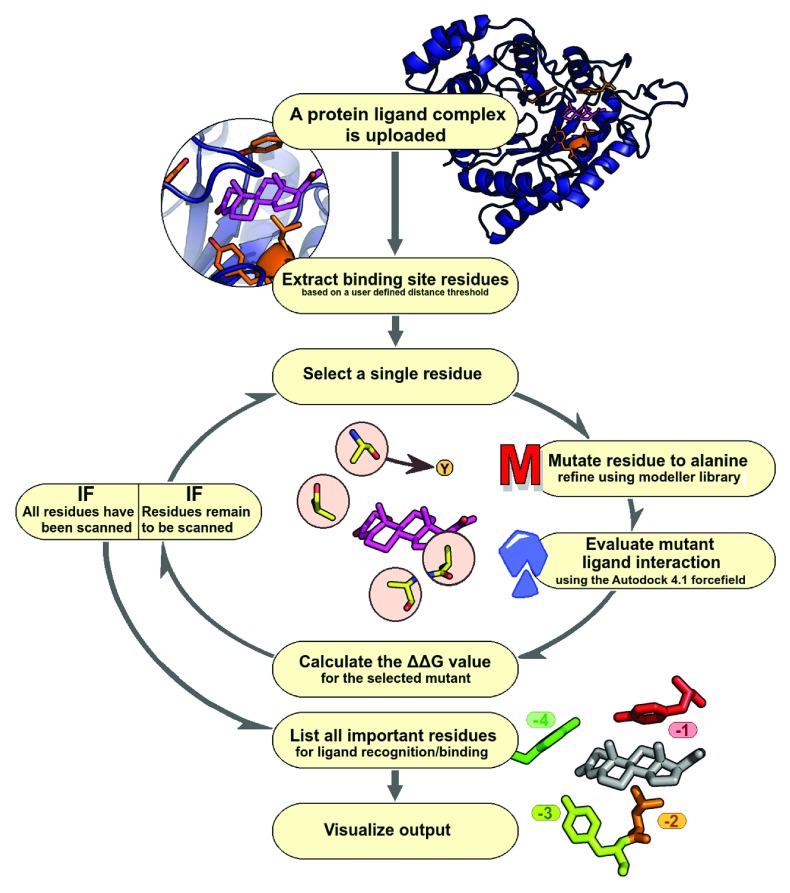
ABS-Scan workflow. Flowchart depicting various steps involved in ABS-Scan.

This workflow allows a user to submit a protein-ligand complex of their interest ( Figure 1). The user is provided with an option of selecting a distance cut-off to define the binding site around a specific ligand for which, *in-silico* alanine scanning mutagenesis is carried out. Once the input parameters are obtained, the Modeller library is used to perform site-specific mutagenesis on all selected residues, coupled with steps of energy minimization ^22^. This consists of initial steps of conjugate gradient (200 iterations with minimum atom shift of 0.001Å), followed by 200 steps of molecular dynamics simulation with steepest descent carried out at different temperatures. The initial restraints for the mutated model are derived from the wild-type protein structure. The analysis and results derived from alanine scanning mutagenesis relies on two assumptions: (a) The introduced point mutation does not drastically change the structure of the protein and (b) the mode of ligand interaction in point mutant is the same in comparison to wild-type complex. Care is taken to ensure that there are no steric clashes between the protein/ligand atoms during the process of minimization. The quality of the protein structures generated is estimated through Discrete Optimized Protein Energy (DOPE) score ^23^, a statistical potential score that is calculated for each of the mutant. This scoring scheme is based on the improved reference consisting of non-interacting atom pairs in a homogenous sphere with radius dependent on sample native structure. The score therefore reflects the feasibility of interactions and the compactness of the modeled structure.

Each mutated structure, will then be scored by using Autodock 4.1 force field ^21^, to calculate the energetics of a protein-ligand complex. The force-field is used here only to score the pose of protein-ligand interaction and no docking is performed. By default, ‘ *check_hydrogens*’ flag is kept ‘on’ while preparing the receptor and Gasteiger charges are used for proteins and ligand. The contribution from a protein residue is determined by difference in interaction score of mutant and wild-type protein (∆∆G value). These results are graphically presented to the user, along with a ranked list of residues in the given site that could be experimentally explored for site-directed mutagenesis. A Jmol applet displays protein-ligand interactions with residues colored according to the computed extents of contribution towards interaction, while a table simultaneously displays inter-molecular energy scores. We also provide a help-section explaining the results along with selected examples.

## Validation and case studies

**Figure 2.**
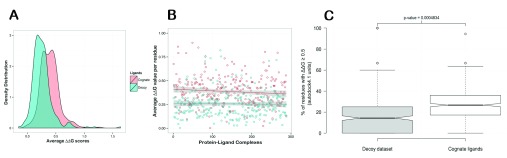
ABS-Scan Sensitivity. ( **A**) The average ∆∆G score per residue distribution from the cognate and decoy protein-ligand complexes of CSAR dataset. ( **B**) The scatter plot displaying the average ∆∆G score for native and the corresponding decoy complexes from the CSAR dataset. ( **C**) Boxplot showing the difference in the % of the residues in the binding site of cognate and decoy complexes having a predicted ∆∆G score ≥ 0.5.

We evaluate the significance of ∆∆G score used to assess the contribution of individual residues at the binding site by systematically analyzing two different datasets. The first dataset was derived from CSAR Community Structure-Activity Resource (CSAR - www.csardock.org/). Decoys in this dataset contain artificial docked complexes of protein with ligands having similar chemical properties to native ligands, but known not to interact with the protein. The protocol could be successfully applied on 288 of 343 protein-ligand native and decoy complexes. The distribution of average ∆∆G scores obtained through ABS-Scan analysis for residues in the binding site for decoy dataset is seen to be different from the native protein-ligand complexes ( Figure 2A & B). An average ∆∆G score of 0.395 was obtained for the native protein-ligand complexes. The second dataset we used to obtain an estimate of ∆∆G score is derived from PDBbind database ^24^ and comprises 195 protein-ligand complexes (PDBbind core dataset). Around 135 of these protein-ligand complexes could be successfully processed using ABS-Scan workflow. In this case, an average ∆∆G score of 0.387 was observed for each mutated residue at the binding site. Hence, to determine the sensitivity of ABS-Scan, a cut-off of 0.5, which is a more stringent value, is chosen. ABS-Scan is seen to effectively discriminate between the decoy and the native complexes of CSAR dataset (p-value ~0.004 calculated with Student’s t-test) in ~67% of the cases (∆∆G ≥ 0.5). This clearly indicates that residues important for ligand interaction can be identified through this protocol ( Figure 2C). The detailed results of ∆∆G scores obtained for each of the mutation produced at the binding site for both these datasets can be accessed from the web-resource - http://proline.biochem.iisc.ernet.in/abscan/validation.

A suitable dataset for validation would be one that reports binding affinities for both wild-type and mutant proteins with same ligand, performed in a uniform experimental environment, for large number of proteins. Although such a dataset exists for protein-protein alanine scanning mutagenesis ^12, 25^, there are none reported for protein-ligand interactions. In order to compare the predictions of ABS-Scan with the experimentally reported alanine-scanning mutations, a methodical search was carried out to mine all the experimental results available in literature on alanine-scanning mutagenesis of residues at the binding site. Advanced search option in PDB was used for this purpose. All the PUBMED extracts were scanned for the term - "alanine scanning". The above search criteria mentioned yielded 126 structure hits with 56 citations. The list of entries obtained, was further pruned to remove biologically irrelevant ligands, metal ions and modified residues. The list of 79 entities/binding sites that we finally obtained can be accessed at http://proline.biochem.iisc.ernet.in/abscan/validation. Alanine scanning could be successfully undertaken for 54 of these structures. On an average, atleast two residues per binding site were predicted to have ∆∆G score ≥ 0.5. The details of the dataset and the ranked lists of residues in the order of their contribution to ligand binding identified for all the complexes is made available to the community - http://proline.biochem.iisc.ernet.in/abscan/validation.

Each of the above experiments involving alanine-scanning mutagenesis reports different mutant evaluation scores. The measures reported to test the fitness of the mutants include various attributes such as K _d_, K _a_, k _cat_/K _M_ (for enzymes), specific substrate **/**product assays *etc.* These measures cannot be normalized to derive values having uniform units for direct comparison. We describe three such examples here, each with different experimentally reported mutant evaluation scores and the predicted ∆∆G values for the same as case studies to highlight the heterogeneity associated with the data.

A study on testosterone binding site of rat 3-alphahydroxysteroid dehydrogenase (PDBID: 1AFS) by Heredia *et al.* ^26^ reports that binding site residue in direct contact with the ligand influences the rate determining step of the enzymatic reaction. In this case, the alanine scanning experiments performed on the residues in the binding site that recognize progesterone and testosterone reports the Kd values. The ABS-Scan analysis performed on 3-alpha hydroxysteroid dehydrogenase in complex with both testosterone and progesterone also predicted the residues W227 (∆∆G score = 1.43; Kd = 10.7±1.2), Y310 (∆∆G score = 1.31; Kd = 9.20±0.94), L54(∆∆G score = 0.5696; Kd = 7.24±0.79) to be important for ligand recognition. A good correlation was observed (0.829 for testosterone and 0.704 for progesterone) between the reported Kd value of the mutants and the corresponding predicted ∆∆G score.

A two-dimensional alanine scanning mutations were performed to understand the structure-function relationship between vitamin-D receptor (PDBID: 1IE9) and vitamin-D analogs by Shimizu *et al.* ^27^. Since there was no structural information available for the analogs complexed to vitamin-D receptor, four of the vitamin-D analogs were docked on the receptor at the vitamin-D native binding site using Rosetta 3.4 docking protocol ^28^. All the poses obtained were analyzed using ABS-Scan to determine the residues crucial for interaction of particular ligands. Since this is a nuclear receptor protein, a transcriptional activity assay was used in original study to evaluate the effect of mutants generated. The effect of each vitamin-D receptor mutant was measured by the downstream transactivation assay that quantifies luciferase activity under the influence of VDR (Vitamin-D Receptor promoter) promoter sequence. In this case, if the mutation affects the binding of ligand, correspondingly the expression of luciferase would reduce by a factor that can be quantified. A good negative correlation was also observed with all the four analogs complexed to vitamin D-receptor and atleast four residues - L233, W286, R274 and H397, important for interaction with all the analogs had ∆∆G score > 0.5. L233 and W286, present in H3 (helix 3) and β sheet are reported to have hydrophobic interactions with B and C rings of the ligand whereas R274 present in H4 (helix 4) is observed to have hydrogen bond interaction with 1α-OH group of the ligand.

A similar study was carried out on human trimethyl-guanosine synthase enzyme (Tgs1) that converts m ^7^G caps (7-methyl guanosine caps) to 2,2,7-trimethylguanosine (TMG) caps. In the original study ^29^ around 37 point mutations were introduced into human Tgs1 (PDBID: 3GDH) to study the interaction profile with mGTP (7-methyl guanosine tri-phosphate) and AdoMet (S-adenosyl methionine). The fitness of mutants generated in this case was evaluated by using the methyltransferase assay that determines the percentage of methylation by quantifying the levels of m ^7^GDP to m ^2,7^GDP. The residues - R807 and K646, reported to be the most affected mutants, are also predicted by ABS-Scan to be essential, with the highest predicted ∆∆G score of 3.63 and 3.39 respectively. These positively charged residues (R807 and K646) are observed to interact with α and β phosphate groups of m ^7^GTP. The π -cation stacking observed between W766 and the m ^7^G was also predicted to be crucial (∆∆G score of 2.66) and correspondingly no methylated products were detected for this mutant through methyl-transferase assay.

The details of the case-studies described above along with the results of the analysis can be accessed on the example section of the web-tool - http://proline.biochem.iisc.ernet.in/abscan/examples.

## Implementation

The web-server was implemented using hypertext preprocessor (PHP). Autodock, Modeller and Pymol libraries have been used for modeling the mutation and evaluating the energetics. Integration of these back-end libraries for presentation as a functional and intuitive user interface is accomplished using Shell, Python, Java, HTML and PHP scripts. The web-server is platform independent and will run on any machine having internet access with browser installed. For the advanced users, a command-line interface in the form of a single python script can be accessed from github repository ( *https://github.com/praveeniisc/ABS-Scan*). The script has been tested on Intel 2.83 GHz quad-core system running 32 bit linux OS(Ubuntu 12.04) with Modeller ^10^, MGL AutodockTools ^30^ & Pymol ( http://pymol.org) installed. For the web-server d3.js library has been used for displaying the plots. Jmol Applet has been used to visualize the protein-ligand interaction.

### Input

The input required for the server is the structure of a protein-ligand complex in PDB format. Users can either provide the four-letter PDBID or upload the PDB structure file of the complex. An option is provided to define the cut-off distance and select the ligand to obtain binding site residues which would be mutated to alanine for evaluating the interaction energetics. A default distance cut-off of 4.5 Å is set to select all the residues whose atoms lie within this distance from any ligand atom. In some the cases, metal ions ^31^ and water molecules are observed to play a crucial role in stabilizing the interactions ^32^. A major problem involved in incorporating the ligand metal ion in ABS-Scan worflow is fixing the charge parameter as metal atoms can have different ionic states (Ex. Fe ^2+^, Fe ^3+^ *etc.*) which is important for evaluating energetics. Enumerating all important structural water molecules involved in the ligand interaction is also highly dependent on the resolution of the crystal structure. Hence, an advanced option is provided to the user for uploading the PDBQT format of the ligand, to account for cases where the ligand contains unusual atom types, metal ions or uses bridge-water molecules for interaction. For practical purposes, the bridge water molecules can be considered to be the part of ligand and these can be incorporated into the pdbqt file of the ligand. As an example, ABS-Scan analysis was carried out on protein lysine methyltransferase (PDB: 3S7B) complexed with S-adenosyl methionine ^33^ through four bridge water molecules. These four bridge-water molecules can be incorporated into the ligand pdbqt file and uploaded with the help of an advanced option provided on the server. The protocol correctly identified GLU135 and ASN182 as significant contributors to ligand binding through formation of water bridges. The output can be accessed through the example section of the web-server.

### Output

**Figure 3.**
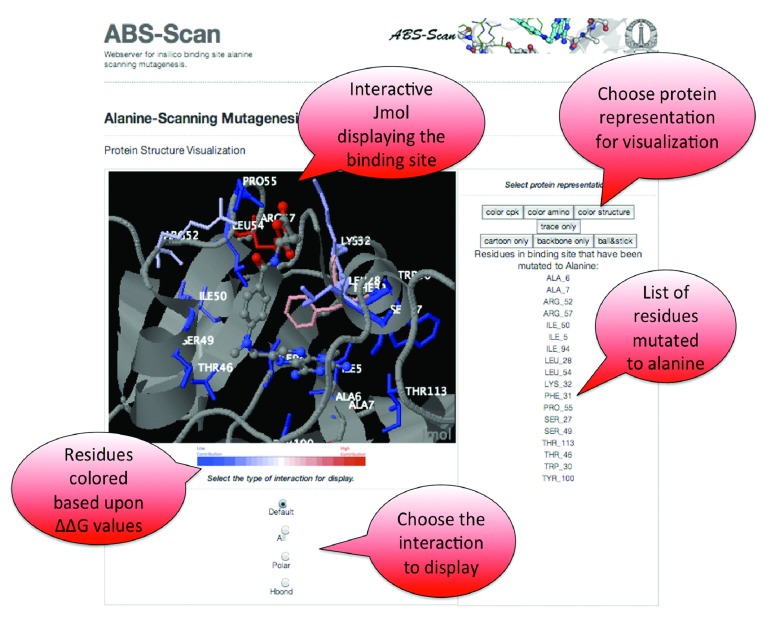
ABS-Scan interactive display. Snapshot explaining the Jmol applet output on the ABScan server. The individual residues are colored in red to blue gradient depending upon the contribution towards the ligand interaction as predicted by ABScan ∆∆G score. Options to visualize the different kinds of interaction - polar, hbonds *etc*. is also provided.

All the results produced by ABScan can be visualized interactively on the web-server. Jmol Applet is used to visualize the contribution of residues towards ligand interaction ( Figure 3).

**Figure 4.**
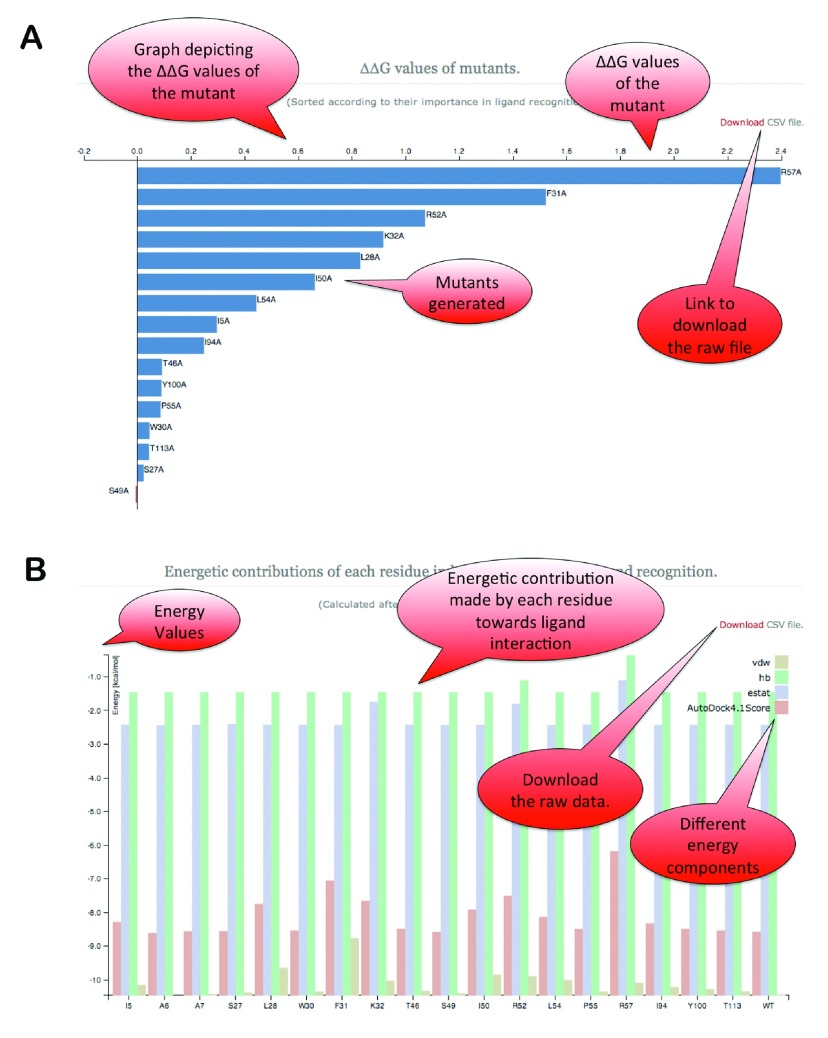
ABS-Scan energy plots. ( **A**) ∆∆G values reported for each of the alanine mutation performed for the residues present at the binding site. The residues are ordered according to their contribution/∆∆G values. ( **B**) The different energy component of autodock interaction score plotted for each of the alanine mutant produced at the binding site.

d3.js library has been utilized to plot the predicted ∆∆G values and subcomponents of the energetic scores reported by Autodock4 ( Figure 4). An option is provided to download publication quality images in SVG/PDF/PNG formats. Twitter bootstrap java library is used for framework development on the webserver. An option is also provided to download the raw files containing individual mutants in PDB format, ∆∆G scores in the raw CSV format along with autodock energy scores.

## Conclusions

ABS-Scan webserver can provide valuable insights on molecular recognition involving protein-ligand interactions. Experimentally determined protein-ligand structures can be studied to understand individual residue contributions towards ligand binding. Modeled complexes can also be submitted to infer the feasibility of the interaction. We believe that ABS-Scan would add one more dimension to the analysis of binding sites in proteins, comparison of various ligand interactions and be of importance to researchers performing ASM studies.

## Software availability

### Software access

http://proline.biochem.iisc.ernet.in/abscan/

### Latest source code

https://github.com/praveeniisc/ABS-Scan

### Source code as at the time of publication

https://github.com/F1000Research/ABS-Scan/releases/tag/V1.0

### Archived source code as at the time of publication

http://dx.doi.org/10.5281/zenodo.12806 ^34^

### Software license

ABS-Scan is licensed under a Creative Commons Attribution-ShareAlike 4.0 International License.
