# Supplementary material for: ABS–Scan: In silico alanine scanning mutagenesis for binding site residues in protein–ligand complex
Source: F1000Res. 2014 Dec 1;3:214. Originally published 2014 Sep 9. [Version 2] doi: 10.12688/f1000research.5165.2 (PMC4319546; doi:10.12688/f1000research.5165.2)
Supplement: Peer review history

# Peer review: Referee response for version 2

**DOI:** 10.5256/f1000research.6207.r11974

**Published:** 2016-01-19

**Authors:** Bernard Offmann

**Affiliations:** 1 Unité Fonctionnalité et Ingénierie des Protéines, Université de Nantes, Nantes, France

No more comments. This version of the manuscript has been improved on several lines and the reply by the authors are acceptable. The paper is approved.

I have read this submission. I believe that I have an appropriate level of expertise to confirm that it is of an acceptable scientific standard.

# Peer review: Referee response for version 2

**DOI:** 10.5256/f1000research.6207.r7324

**Published:** 2015-02-04

**Authors:** Srikrishna Subramanian, Gurmeet Kaur

**Affiliations:** 1 Protein Science and Engineering, CSIR - Institute of Microbial Technology, Chandigarh, India; 2 Institute of Microbial Technology, Chandigarh, India

In their manuscript entitled " ABS–Scan: *In silico* alanine scanning mutagenesis for binding site residues in protein–ligand complex", the authors have discussed the development of an *in silico *workflow for the prediction of important residues for ligand recognition in a given protein-ligand complex. In our opinion, the ABS-Scan tool has been designed and validated satisfactorily. The online web-server is intuitive, fast and easy to use. In the present version of the paper and the software tool, the authors have incorporated the recommendations provided by the previous referees, many of which in our opinion are relevant.

We have the following suggestions.

1. The authors state that 288 of the total 343 protein-ligand complexes from the CSAR dataset, 135 of the 195 protein-ligand complexes from the PDBbind dataset and 54 of the 79 experimental datasets could be processes by ABS-Scan. Although, the authors mention in their responses to referee comments of version 1 of the manuscript, that ABS-Scan rejected some protein-ligand complexes due to “unusual atom types or missing protein/ligand atoms or unusual convention for ligand atoms”, it would probably be helpful for users to determine the kind of protein-ligand complexes that are suitable/unsuitable for prediction using ABS-Scan if authors could discuss this aspect in a sufficiently detailed manner. Was there anything in particular or common in the rejected complexes from the three datasets used for validation? It would be great if they could possibly provide the list of the rejected complexes and the reason for rejection, similar to what they have done for the protein-ligand complexes which were used for validation of the ABS-Scan tool.
2. It is usually seen that short peptide ligands are present as a separate chain in the protein structure files. Particularly for these cases, there is no option in the web-interface of ABS-Scan to enter the other chain as ligand. Can you please elaborate how your software tool handles such protein-ligand complexes?
3. While providing the ΔΔG values for the mutated residues, we think it would be a useful if the ABS-Scan server also provides some indication of evolutionary conservation of the residue. This would allow the users to translate ABS-Scan results from already known or docked protein-ligand complexes to other homologs of the same protein family.

We have read this submission. We believe that we have an appropriate level of expertise to confirm that it is of an acceptable scientific standard.

# Peer review: Referee response for version 2

**DOI:** 10.5256/f1000research.6207.r7122

**Published:** 2014-12-22

**Authors:** Sunando Datta

**Affiliations:** 1 Department of Biological Sciences, Indian Institute of Science Education and Research Bhopal, Bhopal, India

The authors have now  incorporated all the suggestions I made in my 1st review. In fact the revised web application is able to include the solvent molecules. I think that the revised version is now ready to be indexed in PubMed.

I have read this submission. I believe that I have an appropriate level of expertise to confirm that it is of an acceptable scientific standard.

# Peer review: Referee response for version 1

**DOI:** 10.5256/f1000research.5509.r6276

**Published:** 2014-10-17

**Authors:** Bernard Offmann, Stéphane Téletchéa

**Affiliations:** 1 Unité Fonctionnalité et Ingénierie des Protéines, Université de Nantes, Nantes, France

This paper reports an attempt to develop an original tool that simulates alanine scanning mutagenesis to probe residues involved in the process of ligand recognition in proteins.

More precisely, the work describes the development of a work flow that implements known methodologies for homology modeling of alanine single-point mutants of a protein and for molecular docking. Even though, this can be viewed as a methodological paper.

We have some serious concerns regarding this work.

1. The authors claim that they performed a "validation" of  their tool on a dataset that comprises "79 entries" carefully selected from PDB (also cf point 2 below). Their evaluation is based on finding a correlation between docking scores with experimentally determined binding affinities. In their paper, the authors provide evidence of this validation by providing results of "Experimental correlation" for only one example (Figure 2) which relates to binding of rat 3-alpha-hydroxysteroid dehydrogenase (PDB: 1AFS) to testosterone and progesterone. Since they must have it, clearly, the authors should provide their evaluation of this correlation on all "79 entries". I would expect at least that they provide a new Figure 2 that comprises all data points coming from these "79 entries" to sustain their claim and help readers to evaluate the global performance of their tool. They attempted to provide few additional results on their website ( http://proline.biochem.iisc.ernet.in/abscan/validation). It is more confusing because the results provided for the vitamin D receptor (PDB: 1IE9) is not about binding affinities but "translational activity". I'm here suggesting that detailed data for all mutations taken from all "79" entries are provided to the community in the form of a table or downloadable flat or excel-type file.
2. The amount of independent PDB entries in their dataset is not 79. In fact, in some of PDB entries, multiple ligands were observed. Surprisingly, they consider these as separate entries. So their data is redundant with respect to the proteins. 
3. When generating homology models for protein variants, even if these are single point mutants, assessment of the quality of the models is a critical step. Selecting best models may not be that trivial. The authors need to clarify how they implement in their work flow the assessment of the quality of the models and consequently, what criteria they used for selecting the best models (and how many of them) that will be subjected to molecular docking.
4. Regarding the alanine scanning procedure, there are issues regarding the treatment of alanine and proline. They should both be discarded from the alanine scanning protocol: alanine is already present in the structure while proline is not suitable for mutations because of the major protein backbone rearrangements that should be performed to properly mutate it.
5. For such a tool, it is at stake to evaluate its performance using different homology modeling and molecular docking methods. The rational behind the choice of Modeler over other methods like Rosetta is not indicated. Likewise, the reason why Autodock and not Dock etc or even Autodock Vina is not explained.
6. The efficiency of molecular docking using AutoDock is also dependent on the docking protocol used. In such an automated "screen", care should be taken about the preparation of the receptor, the ligand and the grid. For example, are the ligands kept flexible ? In the manuscript, there are no indications about how the authors dealt with this central issue. The authors are encouraged to describe precisely and discuss their docking protocol.
7. According to the AutoDock 4.0 article, the median error range in energy estimation for any protein-ligand evaluation is 1.5-2.0 kcal/mol. In their study, the ∆∆G differences for ligand binding between mutant and native forms of the proteins are far below 2.0 kcal/mol. Thus, it is difficult to rank the mutants. Also, how the authors chose the 0.5 kcal/mol ∆∆G threshold is not clear. There is no discussion how this threshold compares with the intrinsic limits in precision of AutoDock.
8. The definition of ligand in the tool is problematic. In case of oligo or polysaccharides, the carbohydrate residues are erroneously considered separately. For example, in the 1J84 entry from PDB, the carbohydrate-binding module (CBM) is bound to cellotretraose, a 1,4-β-D-glucan composed of four ß-D-glucose residues linked by ß-1,4 osidic linkages. When this PDB entry is submitted to ABS-Scan, it erroneously splits the oligomer into smaller entities that correspond to the chemical IDs of its constituents (BGC 401, 402, 403, 404). This is a serious flaw in their software.
9. While it is common to see people to reuse available codes, the authors do not properly cite the source of their codes they posted on Github and used for providing a complete service to the community: at least 80% of the “alanine_scanning.py” code comes from either MODELLER examples ( http://salilab.org/MODELLER/wiki/Mutate_model) or AutoDock code ( http://mgltools.scripps.edu/api/AutoDockTools/AutoDockTools.Utilities24.compute_AutoDock41_score-pysrc.html). 

We have read this submission. We believe that we have an appropriate level of expertise to state that we do not consider it to be of an acceptable scientific standard, for reasons outlined above.

# Response

**Published:** 2014-11-18

**Authors:** Nagasuma Chandra

**Affiliations:** Indian Institute of Science, India

**We thank the reviewers for their time and effort. There were some useful suggestions, which we have incorporated but do not agree with all the points raised. A detailed point-by-point response is given below.**

1. *The authors claim that they performed a "validation" of their tool on a dataset that comprises "79 entries" carefully selected from PDB (also cf point 2 below). Their evaluation is based on finding a correlation between docking scores with experimentally determined binding affinities. In their paper, the authors provide evidence of this validation by providing results of "Experimental correlation" for only one example (Figure 2)which relates to binding of rat 3-alpha-hydroxysteroid dehydrogenase (PDB: 1AFS) to testosterone and progesterone. Since they must have it, clearly, the authors should provide their evaluation of this correlation on all "79 entries". I would expect at least that they provide a new Figure 2 that comprises all data points coming from these "79 entries" to sustain their claim and help readers to evaluate the global performance of their tool. They attempted to provide few additional results on their website (http://proline.biochem.iisc.ernet.in/abscan/validation). It is more confusing because the results provided for the vitamin D receptor (PDB: 1IE9) is not about binding affinities but "translational activity". I'm here suggesting that detailed data for all mutations taken from all "79" entries are provided to the community in the form of a table or downloadable flat or excel-type file.*
  A suitable dataset for validation would be one that reports binding affinities for both wild-type and mutant proteins with same ligand, performed in a uniform experimental environment, for large number of proteins. Although such a dataset exists for protein-protein alanine scanning mutagenesis for eg., Rosetta alanine scanning), there are none reported for protein-ligand interactions.
  Since no such dataset was available to us, we systematically extracted PDB entries of ligand bound complexes and the corresponding binding sites in them that contained information about experimental alanine-scanning mutagenesis. However, the manner in which the effects of mutagenesis are reported in these differ significantly. While differences in ligand binding strengths (K _a_ or K _d_ values) are reported for some, changes in catalytic efficiencies are reported for some others. For some others, reporter assays are given which indicate capability of the downstream process more qualitatively. Hence it is difficult to perform a systematic comparison from these with the ∆∆G values calculated from our tool in this study. Nevertheless from this dataset, some examples were hand-picked, corresponding primary literature were read and known residue importances obtained, which were then compared with the predicted ones from our tool.  In any case, ABS-Scan analysis has been successfully performed on 54 (the remaining 25 cases were not processed by default steps due to unusual atom types in proteins/ligands) complexes, which provide the extent of contribution to ligand binding of each residue in each  site, in the form of a ranked list of residue-wide ∆∆G values. All this information has been made available to the community, through our webserver -  ( http://proline.biochem.iisc.ernet.in/abscan/validation).
  Besides this, given the lack of systematic reports of experimental data, validation can only be performed to understand the significance of the ∆∆G scores calculated from our tool. For this, we have taken two large datasets (a) protein complexes with native ligands versus decoy ligands from and (b) list of well curated with precise binding site definitions for known protein-ligand complexes used for benchmarking docking algorithms. From both of these, ∆∆G scores are in the range of 0.5 was significant.
  a) A fresh dataset derived from PDB-Bind core dataset consisting of 195 protein-ligand complexes, which has been developed for the purposes of benchmarking docking algorithms (Kim *et al*., 2004, Huang *et al.*, 2008). Of the 195, 135 could be processed successfully for preparation of the protein-ligand complexes for analysis. (The others that could not be included, are likely to contain either unusual atom names or types or missing protein/ligand atoms or unusual convention for ligand atoms and hence could not be processed). 
  b) A dataset of 343 protein-ligand complexes, each with a native and a decoy ligand. 288 structures out of 343 could be successfully evaluated. (Here again the others were omitted due to difficulties in automatic protein/ligand preparation).
  In the process, since ABSscan has been run for all these complexes, information about key contributing residues is generated for each of them. This has been made available through the webserver.  Residue-wise contribution is obtained and presented in a ranked order for each complex, thus providing a ready resource of important residues for ligand binding.
  The results of these can be accessed from the validation section on the webserver – http://proline.biochem.iisc.ernet.in/abscan/validation
2. *The amount of independent PDB entries in their dataset is not 79. In fact, in some of PDB entries, multiple ligands were observed. Surprisingly, they consider these as separate entries. So their data is redundant with respect to the proteins. *
  These reflect independent binding sites (with bound ligands).  As can be expected, some proteins have multiple sites with different ligands, making it necessary to consider them separately. Hence 79 sites are unique and come from 46 PDB entries. In the original manuscript, the dataset of 79 was never meant to reflect ‘ **unique PDB entries’**. In any case we refer to them now as ‘binding site entities’ to reflect this more clearly.
3. *When generating homology models for protein variants, even if these are single point mutants, assessment of the quality of the models is a critical step. Selecting best models may not be that trivial. The authors need to clarify how they implement in their work flow the assessment of the quality of the models and consequently, what criteria they used for selecting the best models (and how many of them) that will be subjected to molecular docking.*
  Model quality has been considered as part of the modelling pipeline itself. Given the scale of the study, it is practical to generate one model for each mutant, but care is taken to ensure that it is optimal and free of errors in terms of bad contacts or atomic clashes. The optimization protocol used consists of 200 iterations of conjugate gradient, followed by molecular dynamic simulation for 4fs and simulated annealing with 200 iterations at different temperatures (This is the default protocol suggested in Model_mutate.py of Modeller - http://salilab.org/modeller/wiki/Mutate%20model). The initial restraints for generation of the model is derived from the wild-type structure itself. Assumptions necessary for modelling point mutations introduced through alanine-scanning mutagenesis protocol at the binding sites are that (a) they are unlikely to change the overall structure of the protein drastically and (b) the ligand moiety roughly retains the same conformation in comparison with the wild-type complex to interact with the mutated structure.
  Since modelling protocols have been well established for a long time now, we did not see the need for adding this information explicitly in the original MS.  In any case, based on the reviewers suggestion,  this information has been added to the revised version. Normalized DOPE scores are reported for both the native and mutant structures. DOPE refers to ‘Discrete Optimized Protein Energy’ and is a statistical potential which checks for the feasibility of the observed interactions. Protein structures with lower DOPE scores (typically in negative range -1.5 to -2.5 for experimentally solved structures) can be considered to be of good quality ( Shen and Sali *., *2006). 
4. *Regarding the alanine scanning procedure, there are issues regarding the treatment of alanine and proline. They should both be discarded from the alanine scanning protocol: alanine is already present in the structure while proline is not suitable for mutations because of the major protein backbone rearrangements that should be performed to properly mutate it.*
  This required addition of simple screens to filter out these residues from consideration for alanine scanning, which has been done. Changes have been made to both the source code and the web-tool now. Glycine mutations are also filtered out. 
5. *For such a tool, it is at stake to evaluate its performance using different homology modeling and molecular docking methods. The rational behind the choice of Modeler over other methods like Rosetta is not indicated. Likewise, the reason why Autodock and not Dock etc or even Autodock Vina is not explained.*
  The goal of our study is not to develop a modelling algorithm or a new parameter for building models. The most widely used tool for homology modelling – Modeller, which we have currently included in the workflow, has about 1500 citations. Currently there are more than 50 tools for homology modeling -( http://en.wikipedia.org/wiki/List_of_protein_structure_prediction_software) and roughly the same number of tools for protein-ligand docking ( http://en.wikipedia.org/wiki/Docking_%28molecular%29). The precise reason for choosing ‘Modeller’ or ‘Autodock’ is perhaps because of our own experience in using these tools along with availability of extensive documentation, tutorials and ease of implementation.  Moreover, both these libraries had python bindings available and hence could be merged into a single script using python. In future, we plan to develop a pymol plugin for the same. 
  A simple bash script for processing the protein-ligand complex to determine the interaction energy using ROSETTA force fields has also been included in the github repository. This again, is only for the advanced users and we might incorporate it in the future versions of the pipeline.
6. *The efficiency of molecular docking using AutoDock is also dependent on the docking protocol used. In such an automated "screen", care should be taken about the preparation of the receptor, the ligand and the grid. For example, are the ligands kept flexible? In the manuscript, there are no indications about how the authors dealt with this central issue. The authors are encouraged to describe precisely and discuss their docking protocol.*
  We would like to clarify here that there is no docking performed in the whole exercise. We only score the complex in the given conformation using the force fields. By default, through prepare_receptor4.py and prepare_ligand4.py Gasteiger charges and polar hydrogens are added while evaluating the interaction energy.  This has been mentioned in the manuscript:
  “Each mutated structure, will then be scored by using Autodock 4.1 force field, to calculate the energetics of a protein-ligand complex. The contribution from the residue is then determined by calculating the difference in interaction score of the mutant and the wild-type protein (∆∆G value).”
7. *According to the AutoDock 4.0 article, the median error range in energy estimation for any protein-ligand evaluation is 1.5-2.0 kcal/mol. In their study, the ∆∆G differences for ligand binding between mutant and native forms of the proteins are far below 2.0 kcal/mol. Thus, it is difficult to rank the mutants. Also, how the authors chose the 0.5 kcal/mol ∆∆G threshold is not clear. There is no discussion how this threshold compares with the intrinsic limits in precision of AutoDock.*
  The median error range of the energy estimation reported in AutoDock 4.0 article is for the total ∆G score between the experimental and predicted values, whereas in this case it is for individual residue contributions. The distribution of the ∆∆G values obtained for the decoy and cognate ligands from the CSAR dataset ( http://www.csardock.org/) was used to define a cut-off of 0.5. This has also been validated on PDBbind core dataset ( http://www.pdbbind-cn.org/). Figures 3A and 3B have been added along with explanations in the manuscript.
  We believe that intrinsic limits on precision of Autodock scoring would not be a major concern as both the wild type and the mutant are evaluated using the same scoring scheme and the cut-off has been chosen on basis of native protein-ligand complexes in CSAR and PDBbind datasets.
8. *The definition of ligand in the tool is problematic. In case of oligo or polysaccharides, the carbohydrate residues are erroneously considered separately. For example, in the 1J84 entry from PDB, the carbohydrate-binding module (CBM) is bound to cellotretraose, a 1,4-β-D-glucan composed of four ß-D-glucose residues linked by ß-1,4 osidic linkages. When this PDB entry is submitted to ABS-Scan, it erroneously splits the oligomer into smaller entities that correspond to the chemical IDs of its constituents (BGC 401, 402, 403, 404). This is a serious flaw in their software.*
  This is not really a 'problem' and is an established work-around to avoid long computation and hence long waiting time for the user. All this does is to split peptides or oligosaccharides into individual moieties (typically for a peptide, each amino acid is considered as a moiety and for an oligosaccharide, each monosaccharide is considered as a moiety), as per the convention currently followed by PDB.  How can this be a ‘serious flaw’? It does not, in any manner, influence the results. Many other tools for protein-ligand interaction analysis such as LPC (Ligand-protein contacts, Ligplot+, Ligplus *etc.)* also track ligands through such residue identifiers.
  However, an advanced option has now been added to provide the range of the ligand residue numbers to be considered as a single moiety during the entire protocol. For example, now a residue range 401-404 can be provided for 1J84 instead of a single residue number to consider the whole oligocomplex as single ligand. The script has also been accordingly modified in github.
9. *While it is common to see people to reuse available codes, the authors do not properly cite the source of their codes they posted on Github and used for providing a complete service to the community: at least 80% of the “alanine_scanning.py” code comes from either MODELLER examples (http://salilab.org/MODELLER/wiki/Mutate_model) or AutoDock code (http://mgltools.scripps.edu/api/AutoDockTools/AutoDockTools.Utilities24.compute_AutoDock41_score-pysrc.html).*
  We have indeed already cited all the tools used in the manuscript to which source codes are linked. In any case, these references are now highlighted in the source code also. Both Autodock and Modeller are released under the GNU public license, making their source code freely usable to all interested parties.  Moreover, these are the primary source and codes are not extracted from any third-party tools. The purpose of putting it on Github is to be completely open about the details of the protocol and make our work fully accessible to anyone interested.   
  We would again like to remind the reviewer here that source-code is used only by an advanced user. The reviewer may be aware of the time and effort involved in producing a web-application interface that is embedded with visualization features. This has been done with the belief that it will save precious time for researchers who do not have the expertise or the interest in installation and handling command-line interfaces for such tools. We initially proposed this as a web-tool, but since that section is no longer available in *F1000Research*, we submitted it as a software tool.

# Peer review: Referee response for version 1

**DOI:** 10.5256/f1000research.5509.r6262

**Published:** 2014-09-30

**Authors:** Sunando Datta

**Affiliations:** 1 Department of Biological Sciences, Indian Institute of Science Education and Research Bhopal, Bhopal, India

The manuscript 'ABS–Scan: *In silico *alanine scanning mutagenesis for binding site residues in protein–ligand complex' reports development of a web server for performing *in silico* alanine scanning mutations for studying protein-small molecule interactions. It further validates the tool by taking a list of already published Alanine scanning data along with the X-ray crystallographic structures of the relevant protein-ligand complexes. ABS-Scan provides a user-friendly web interface and will be very much useful for experimentalists to assess the outcome of mutations designed to study protein-ligand (small molecule) interactions. Overall the web tool is well explained in the manuscript.

My main concern is the method for energy calculations, authors used to predict the individual contribution upon mutation. It does not include waters and metals. As it is well established that many of the protein-ligand interactions are water mediated and therefore water plays very important role in determining the specificity as well as affinity. Authors could use energy function which includes waters and metals as well otherwise the current version could only be used for protein-ligand complexes in which water/metal atoms have been shown to play any role in stabilizing the ligand in the binding pocket.

Thus in my opinion, I think it could be indexed with inclusion of an updated energy function. Alternatively, its sole applicability for protein-ligand interaction without involvement of solvent molecules should be mentioned in the conclusion.

I have read this submission. I believe that I have an appropriate level of expertise to confirm that it is of an acceptable scientific standard, however I have significant reservations, as outlined above.

# Response

**Published:** 2014-11-18

**Authors:** Nagasuma Chandra

**Affiliations:** Indian Institute of Science, India

We thank the reviewer for going through our manuscript and finding the work useful. These are indeed valid points and have been addressed in the revised version. 

Bridge water molecules do play an important role in the protein-ligand interactions. One has to take into account the resolution of the protein structure to determine the confidence of the placed water molecules. Hence an advanced option is provided wherein these water molecules when present at the site can be considered to be a part of the corresponding ligand moiety. The user can upload his/her own pdbqt file for the ligand with the appropriate water molecules added to it.  An example of protein lysine methyltransferases complexed with S-adenosyl methionine has been described in the manuscript. The corresponding pqbqt file of the ligand can be downloaded from the example section. The results of these can also be accessed from example section of the web-server.
